# The *C. elegans* Casein Kinase II is associated with meiotic DNA in fertilized oocytes

**DOI:** 10.17912/micropub.biology.000583

**Published:** 2022-06-06

**Authors:** Nahyun Yim, Jeffrey C. Medley, Mi Hye Song

**Affiliations:** 1 Department of Biological Sciences, Oakland University, Rochester, MI, USA

## Abstract

By using CRISPR/Cas9 genome-editing, we have generated epitope-tagged KIN-3 and KIN-10 expressing strains at the endogenous C-terminal loci in *Caenorhabditis elegans* . We observed that both the catalytic (KIN-3::V5) and regulatory (KIN-10::2xMyc) subunits of the Casein Kinase II (CK2) holoenzyme complex are associated with meiotic DNA, enriched in the midvalent rings during meiotic divisions in fertilized *C. elegans* oocytes.

**Figure 1. Both KIN-3 and KIN-10 are associated with meiotic DNA in fertilized C. elegans oocytes:**
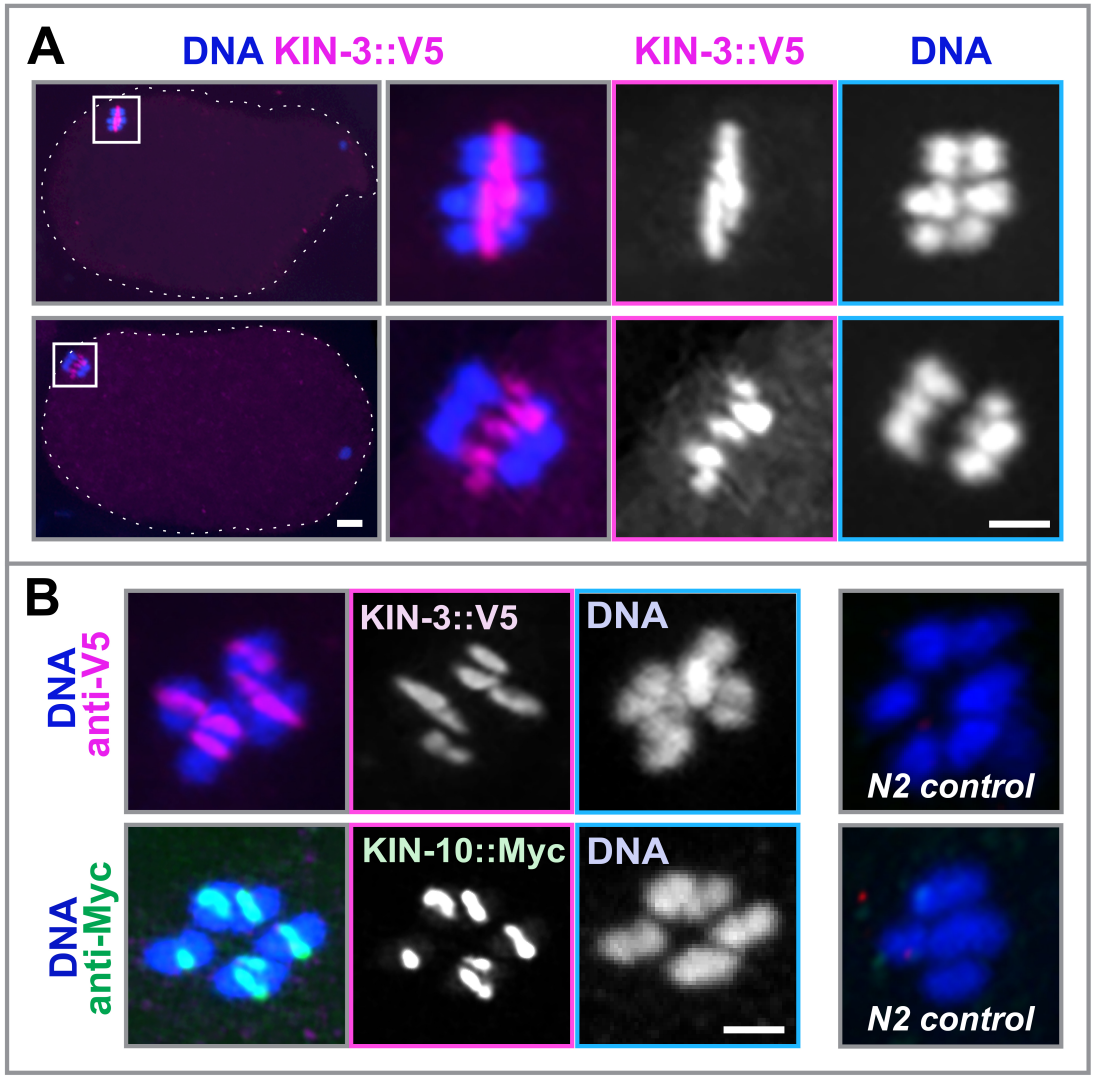
Immunofluorescence images of the fertilized oocytes, stained with DAPI (blue) and α-V5 (magenta) to detect KIN-3::V5 **(A, B)** or α-Myc (green) to detect KIN-10::2xMyc ( **B** ). ( **B** ) α-V5 and α-Myc specifically detect KIN-3::V5 and KIN-10::2xMyc, respectively, but show no signal in N2 (wild-type) controls. Scale bars, 2.5 µm. &nbsp;

## Description

The kinase Casein Kinase II (CK2), a tetrameric holoenzyme, consists of two catalytic (CK2α) and two regulatory (CK2β) subunits (Niefind et al., 2009). The *C. elegans* catalytic and regulatory subunits are encoded by *kin-3* and *kin-10* , respectively (Hu and Rubin, 1990, 1991). We used CRISPR/Cas9 genome-editing to generate epitope-tagged KIN-3::V5 and KIN-10::2xMyc expressing strains at the endogenous C-terminal loci. By staining early embryos with commercially available epitope antibodies, we observed the localization of KIN-3::V5 and KIN-10::2xMyc in fertilized *C. elegans* oocytes. During meiosis in fertilized *C. elegans* oocytes, both the catalytic (KIN-3) and regulatory (KIN-10) subunits of the CK2 holoenzyme are associated with meiotic DNA, enriched around the center of the bivalent, referred to as the ring complex (Dumont et al., 2010; Davis-Roca et al., 2016). It has been shown that AIR-2/Aurora B and KLP-19 localize to the ring complexes associated with meiosis I bivalent and meiosis II chromosomes. Both AIR-2 and KLP-19 are required for proper chromosome segregation during *C. elegans* meiosis (Dumont et al., 2010; Davis-Roca et al., 2016). The close association of KIN-3 and KIN-10 with meiotic DNA suggests that CK2 kinase activity might influence chromosome organization and segregation during meiotic divisions in the *C. elegans* oocyte. In support of this, previous work has reported that depletion of CK2 results in polar body extrusion failure and extra DNA, likely due to meiotic errors in fertilized *C. elegans* oocytes (Medley et al., 2017). A study in porcine oocytes has also shown that CK2 localizes to meiotic chromosomes and that CK2 activity is required for normal meiotic progression (ShiYang et al., 2020). Thus, CK2 function during meiotic division appears to be evolutionarily conserved.

## Methods

***C. elegans* Culture:** All strains were derived from the wild-type Bristol N2 strain and maintained on MYOB plates seeded with *Escherichia coli* OP50 at 20°C.

**Immunostaining and Confocal Microscopy:** Immunofluorescence and confocal microscopy were performed as described (Medley et al., 2017). For immunostaining, the following primary and secondary antibodies were used at 1:3000 dilutions: α-Myc (GenScript, # A00704), α-V5 (GenScript, # A01724), and Alexa Fluor 488 and 568 secondary antibodies (ThermoFisher, #A11001, A11004, A11006, A11034, A11036). Confocal microscopy was performed using a Nikon Eclipse Ti-U microscope equipped with a Plan Apo 60×1.4 NA lens, a Spinning Disk Confocal (CSU X1), and a Photometrics Evolve 512 camera. MetaMorph software (Molecular Devices, Sunnyvale, CA, USA) was used for image acquisition and Adobe Photoshop/Illustrator 2022 for image processing.

**CRISPR/Cas9 Genome Editing:** For genome editing, we used the co-CRISPR technique described previously (Arribere et al., 2014, Paix et al., 2015). To design crRNA, we used the CRISPOR webserver (crispor.tefor.net; Concordet and Haeussler, 2018). Animals were microinjected with a mixture of commercially available SpCas9 (IDT, Coralville, IA) and custom-designed oligonucleotides (IDT, Coralville, IA) including crRNAs at 0.4–0.8 µg/ml ( *kin-3:* 5'-AUUUUAAGCGCCGUCAAUUU-3', *kin-10* : 5'-GGAGGACAAUUCAAUAAUUA-3') tracrRNA at 12 µg/ml, and single-stranded DNA oligonucleotides at 25–100 ng/ml. After injection, we screened for *dpy-10(cn64) II/+* rollers in F1 progeny and genotyped F2 for the epitope-tag insertion. The genome editing was verified by Sanger Sequencing (GeneWiz, South Plainfield, NJ). All the *C. elegans* strains generated in this study produce nearly 100% viable progeny.

Single-stranded DNA oligonucleotides homologous repair templates (IDT, Coralville, IA) for genome editing were as follows.

KIN-3::V5 tag at the C-terminus (5'-3'): CATCGAATTCCGCTTCTTCTCAATCCTCCGATGCTAAAATTGACGGCGCTGGAGGTTCCGGTGGTTCTGGTGGATCC ***GGTAAGCCTATCCCAAATCCTTTGTTGGGTCTGGACTCCACG*** TAAAATTTCTTTCTATTTTTTTTTTAATTTTCCTG

KIN-10::2xMyc tag at the C-terminus (5'-3'): CAAAACAACACGACTCCAGCCGGGCAACAATCTGGCGGCCAGTTCAACAACTATGGTCTCGGTGGCTCTGGTGGAAGTGGAGGCTCA ***GAACAAAAACTGATATCTGAAGAAGACCTTGAGCAGAAGTTGATTAGTGAGGAGGATCTT*** TGAGCCACTTTCTTCCTTATTTTTGTTTTGATTTC

## Reagents

**N2: wild-type (CGC),** MTU137: *kin-3(mhs464[KIN-3::V5]) I* (This study), MTU598: *kin-10(mhs688[KIN-10::2xMyc]) I* (This study)
